# Intra-host symbiont diversity in eastern Pacific cold seep tubeworms identified by the *16S-V6* region, but undetected by the *16S-V4* region

**DOI:** 10.1371/journal.pone.0227053

**Published:** 2020-01-15

**Authors:** Corinna Breusing, Maximilian Franke, Curtis Robert Young

**Affiliations:** 1 Monterey Bay Aquarium Research Institute, Moss Landing, CA, United States of America; 2 National Oceanography Centre, Southampton, England, United Kingdom; 3 Max Planck Institute for Marine Microbiology, Bremen, Germany; CNRS-UPS, FRANCE

## Abstract

Vestimentiferan tubeworms are key taxa in deep-sea chemosynthetic habitats worldwide. As adults they obtain their nutrition through their sulfide-oxidizing bacterial endosymbionts, which are acquired from the environment. Although horizontal transmission should favor infections by various symbiotic microbes, the current paradigm holds that every tubeworm harbors only one endosymbiotic *16S* rRNA phylotype. Although previous studies based on traditional Sanger sequencing have questioned these findings, population level high-throughput analyses of the symbiont *16S* diversity are still missing. To get further insights into the symbiont genetic variation and uncover hitherto hidden diversity we applied state-of-the-art *16S-V4* amplicon sequencing to populations of the co-occurring tubeworm species *Lamellibrachia barhami* and *Escarpia spicata* that were collected during E/V *Nautilus* and R/V *Western Flyer* cruises to cold seeps in the eastern Pacific Ocean. In agreement with earlier work our sequence data indicated that *L*. *barhami* and *E*. *spicata* share one monomorphic symbiont phylotype. However, complementary CARD-FISH analyses targeting the *16S-V6* region implied the existence of an additional phylotype in *L*. *barhami*. Our results suggest that the *V4* region might not be sufficiently variable to investigate diversity in the intra-host symbiont population at least in the analyzed sample set. This is an important finding given that this region has become the standard molecular marker for high-throughput microbiome analyses. Further metagenomic research will be necessary to solve these issues and to uncover symbiont diversity that is hidden below the *16S* rRNA level.

## Introduction

Vestimentiferan tubeworms (Polychaeta; Siboglinidae) are among the foundation species in deep-sea chemosynthetic communities worldwide. The 10 currently recognized genera in this well-defined siboglinid clade [1] are commonly partitioned into subgroups based on habitat types [2] and evolutionary relationships: the hydrothermal vent genera (*Riftia*, *Ridgeia*, *Tevnia*, *Oasisia*, *Alaysia*, *Arcovestia*) and the hydrocarbon seep genera (*Lamellibrachia*, *Escarpia*, *Paraescarpia*, *Seepiophila*). Vestimentiferans living at vents experience frequent ecological disturbances and unpredictable changes in physicochemical conditions, which has led to the evolution of 'weedy' species with short lifespans and fast growth rates. In contrast, tubeworms living in seeps experience relatively stable habitat conditions with more constant and less toxic fluid flows, which has led to long-lived and slow-growing species [2]. Nonetheless, some of the putative 'vent' species have been found at cold seeps and some of the 'seep' species have been found at vents, whale falls and even shipwrecks [2–6]. Thus, a strict ecological separation of 'vent' and 'seep' tubeworm genera does not appear to exist.

All adult vestimentiferans lack a functional digestive system and must therefore acquire their nutrition entirely from chemosynthetic bacterial endosymbionts that inhabit specialized cells within a complex, anatomically adapted organ of the tubeworm trunk, the trophosome [7, 8]. These bacteria oxidize reduced sulfur compounds to gain energy for the production of organic matter, part of which is shared with the tubeworm host. Studies on *Riftia pachyptila* indicate that tubeworms produce aposymbiotic larvae that acquire their symbionts horizontally via infection by free-living bacteria from the local environment in which the larvae settle [9]. Penetration of the larval epidermis by infecting bacteria triggers metamorphosis to a gutless juvenile stage and initiates a profound renewal of the skin, thereby preventing further symbiont infections. After the death of the tubeworm the symbionts are released to the free-living population and regain the potential to infect new hosts, which ensures the persistence of this partnership in subsequent generations [10]. The potential for multiple infections by different locally adapted bacterial strains creates opportunities for selective enrichment of potentially beneficial strains, but it decouples dispersal of the host larvae from dispersal of the symbionts, thereby increasing the risk of failing to acquire a suitable symbiont [6, 11, 12].

Infectious environmental acquisition, as seen in *Riftia*, is expected to result in heterogeneous symbiont populations within host individuals [6]. Nevertheless, most *16S* rRNA studies to date suggest that individual tubeworms harbor symbiont populations composed of a single gammaproteobacterial phylotype [2, 6, 12, 13], but see [14–18]. Tubeworm symbionts form a monophyletic clade that is subdivided into two major habitat-specific phylotypes. The seep tubeworms (e.g., *Lamellibrachia* and *Escarpia*) host *16S* rRNA phylotype I, which comprises three subgroups that show different depth distributions [12]. By contrast, vent tubeworms (e.g., *Riftia* and *Tevnia*) host the closely related phylotype II, *a*.*k*.*a*. *Candidatus Endoriftia persephone* [2, 6, 13]. A third, distantly related phylotype group was recently described from low-diffuse vents in the Caribbean Sea [19]. A number of studies [14–18] questioned the lack of intra-host variation of the tubeworm symbionts and provided evidence that distinct phylotypes and subtypes can exist in a single tubeworm individual.

The diversity of symbiont populations within individual hosts and among geographically disjunct vent and seep localities is likely to be underestimated. Although genomic references for seep and vent endosymbionts are now available [13, 19–22], population genetic studies of tubeworm symbionts have relied on direct sequencing or cloning of PCR products [6, 14–18], methods that are biased towards the most abundant bacterial types in mixed symbiont infections [18, 23]. Given these limitations Zimmermann et al. [18] argued that advanced molecular techniques would be needed to uncover symbiont variation at the *16S* level. Such methods would probably also be needed to reveal specificity between host species and symbiont phylotypes. A few studies indicated that some degree of host-symbiont specificity might exist in vestimentiferan symbioses [6, 12, 14]. For example, co-distributed tubeworm species do not always take up identical symbiont subtypes, but small-scale patchiness in local environments might account for these results [15].

**Fig 1:**
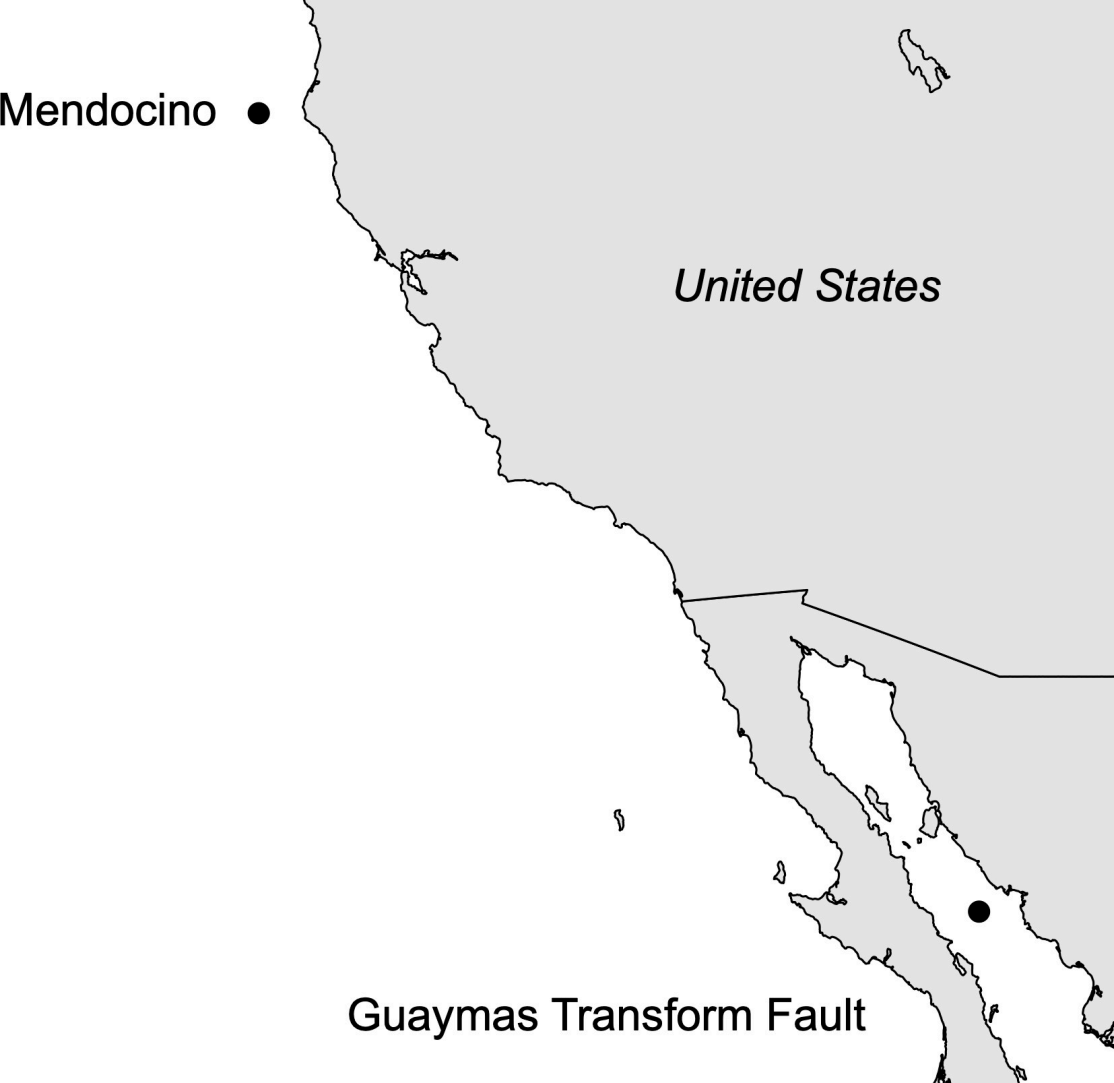
Tubeworm sampling localities in the eastern Pacific Ocean.

In the present study we used high-throughput *16S-V4* amplicon sequencing and complementary CARD-FISH analyses in co-occurring eastern Pacific populations of the seep-associated tubeworm species *L*. *barhami* and *E*. *spicata* (Fig 1; Table 1) to shed more light on symbiont diversity and specificity in vestimentiferan tubeworms. We chose this gene region for our analyses because (1) it is the most commonly used fragment for bacterial diversity analyses and (2) publicly available *16S* sequences from tubeworm symbionts contain a few single nucleotide polymorphisms in the *V4* region. Our assumption was that this variation is actually higher and has so far been obscured by low-throughput methods. In contrast to these expectations, our sequencing data indicated that the *V4* hypervariable region is monomorphic between and within *L*. *barhami* and *E*. *spicata* hosts, while additional CARD-FISH in the *V6* region provided evidence for phylotypic diversity.

**Table 1:** Sampling information for the investigated cold seep sites.

Locality	Latitude	Longitude	Depth (m)	Dive #*	N^§^	Year	Host^†^	Analysis
Mendocino	40°21'N	125°13'W	1578	T: 448	12	2002	L	Amplicon
Guaymas Transform Fault	27°34'N 27°34'N 27°30'N 27°30'N	111°26'W 111°26'W 111°40'W 111°40'W	1778 1778 1722 1722	T: 548 T: 548 H: 1651 H: 1651	12 9 3 3	2003 2003 2017 2017	E L E L	Amplicon Amplicon CARD-FISH CARD-FISH

*Submersibles: H = *Hercules*, T = *Tiburon*

§N: Sample size

†Host: E = *Escarpia spicata*, L = *Lamellibrachia barhami*

## Methods

### Sample collection and DNA extraction

Tubeworm specimens were collected with remotely operated vehicles (ROVs *Tiburon* and *Hercules*) from two widespread locations in the eastern Pacific Ocean (Table 1; Fig 1). Permissions for animal collections during the R/V *Western Flyer* 2002 cruise in US territorial waters were not required. Sampling permits for seeps in the Gulf of California during the R/V *Western Flyer* 2003 and E/V *Nautilus* 2017 (NA090) cruises were obtained by the Monterey Bay Aquarium Research Institute and the Ocean Exploration Trust from Mexico's Secretariat of Foreign Affairs (SRE: DAN-00254, EG0072017), the Secretariat of Environment and Natural Resources (SEMARNAT: SGPA/DGVS/5152) and the National Aquaculture and Fishing Commission (CONAPESCA: 13103.613-03/0057, PPFE/DGOPA-010/17) where necessary. As soon as possible after recovery of the vehicles, tubeworms were removed from their tubes, dissected on individual dishes and frozen at –80°C. Only individuals with intact trophosomes were considered for analysis. For *16S* amplicon sequencing DNA from 21 *L*. *barhami* and 12 *E*. *spicata* individuals was extracted at the Monterey Bay Aquarium Research Institute (Moss Landing, CA, USA) with the DNeasy Blood & Tissue kit (Qiagen, Hilden, Germany). We followed the manufacturer's instructions, except for adding a second elution step to increase DNA yield. Because symbiont distributions can vary across the length of the trophosome [18], we sampled serial tissue sections and used the homogenates for DNA extractions. The PowerClean Pro DNA clean-up kit (Mo Bio, Carlsbad, CA, USA) was used to remove contaminants that might inhibit PCR. For CARD-FISH analyses trophosome pieces of three *L*. *barhami* and three *E*. *spicata* individuals from the E/V *Nautilus* 2017 cruise to the Guaymas Transform Fault were fixed in PFA overnight, washed three times in PBS and then stored in PBS:ethanol. The PFA fixed specimen were not used for DNA analyses.

### Host species identification

Mitochondrial *COI* sequences were used to verify the tubeworm species identifications that were initially based on morphology while on shipboard. A ~700 bp fragment of the *COI* gene was amplified on a Veriti thermal cycler (Applied Biosystems, CA, USA) using 10 *p*mol of primers jgLCO1490 and jgHCO2198 [24], 12.5 μl AmpliTaq Gold Fast Master Mix (Thermo Fisher Scientific, Foster City, CA, USA) and >20 *n*g of DNA adjusted to a total volume of 25 μl with PCR grade water. Negative controls without template were included on each PCR plate to check for sample contamination. Cycling and sequencing protocols followed [25]. Sequence analysis was performed with GENEIOUS v9.1.8 (http://www.geneious.com/) as described in [26]. All sequences were compared to the NCBI Nucleotide Collection with MEGABLAST to determine species identities. POPART v1.7 (http://popart.otago.ac.nz) was used to draw haplotype networks based on the median joining algorithm.

### Symbiont *16S* amplicon sequencing

Barcoded amplicon libraries targeting a ~250 bp fragment of the hypervariable *V4* region of the *16S* rRNA gene were prepared using the primer pair 515f/806r [27] in a single PCR step. PCR amplifications were done in triplicate with the AmpliTaq Gold Fast Master Mix (Thermo Fisher Scientific, Foster City, CA, USA) in a volume of 25 μl. We used the same PCR protocol as [27], but decreased the number of cycles from 35 to 26 to reduce PCR induced mutations and chimera generation. Three negative controls were included to check for cross-contamination. After cleanup with AMPure XP beads (Beckmann Coulter, Brea, CA, USA), final libraries were quantified with the Quant-iT PicoGreen dsDNA assay (Thermo Fisher Scientific, Eugene, OR, USA) and then mass normalized to 240 ng per sample. Pooled libraries were sent to SeqMatic (Fremont, CA, USA) for 2x250 bp paired-end sequencing on one lane of an Illumina HiSeq2500 platform.

### Sequence analysis and identification of operational taxonomic units (OTUs)

Demultiplexed paired-end reads were quality checked in FASTQC v0.11.5 [28] and then adapter clipped with TRIMMOMATIC v0.38 [29]. Reads were merged, filtered, dereplicated and clustered with USEARCH v11 [30]. In addition, we tried the QIIME1 and QIIME2/DADA2 OTU clustering pipelines [31]. The minimum %id of alignment and the maximum number of mismatches during merging were set to 80 and 10, respectively, while the merging length was constrained to 230–270 bp. Merged reads that had an error rate >0.001 and were shorter than 230 bp after truncation at a base quality threshold of 20 were discarded. Dereplication, denoising and clustering of reads into ZOTUs was performed with the *fastx_uniques* and *unoise3* commands. All merged reads were then mapped to the clustered sequences with the *otutab* command to generate an OTU table. Taxonomic classification was performed in QIIME2 (https://qiime2.org) with the silva-132-99-515-806-nb-classifier. Samples with less than 1000 reads, OTUs with less than 100 reads as well as singletons were filtered from the OTU table. This approach recovered one main symbiont OTU in all samples, which we hereafter call Seep symbiont 1. To identify whether this OTU could be separated into different genotypes we applied the OLIGOTYPING v2.0 pipeline [32] on the merged and padded symbiont reads.

### CARD-FISH

To verify the occurrence of Seep symbiont 1 in the trophosomes of *L*. *barhami* and *E*. *spicata* we designed specific horseradish peroxidase (HRP) labeled oligonucleotide probes for the *V4* region of the *16S* rRNA. Unlabeled helper probes flanking the target regions were designed to open up the *16S* rRNA secondary structure and to make the binding sites accessible, while unlabeled competitor probes were used to avoid unspecific hybridizations of the HRP-probes (Table 2). A 0–60% formamide series with 10% increments was performed to identify the formamide working concentrations for the different oligonucleotide probes. Clear signals were only observed at 20% formamide. The general eubacterial probe EUB338I-III [33] and the nonsense probe NON338 [34] were used as positive and negative controls, respectively. To further investigate symbiont variability in the trophosome, we used the probe L_mars1 [18], which targets the hypervariable *V6* region. An alignment showing the binding specificity of the Seep symbiont 1 and L_mars1 probes is given in S1 Fig. All probes were used on the same sample preparations. Although we processed each of the six PFA-fixed samples for CARD-FISH, the *E*. *spicata* trophosome pieces were mostly lost during processing of the microscope slides, so that we focused our analyses on *L*. *barhami*. Optimal tissue sections and preservations were obtained for *L*. *barhami* #45 and we therefore performed essentially all double hybridizations on this individual.

**Table 2:** CARD-FISH probe sequences.

Name	Sequence (5’– 3’)	Reference
Probe_Seep_symbiont_1	GTCAGTGTTGGTCCAGGA	This study
Helper1_Seep_symbiont_1	AGTCGCCTTCGCCACTGA	This study
Helper2_Seep_symbiont_1	ACGCTTTCGCACCTCAGC	This study
Comp1_Seep_symbiont_1	GTCAGTGCTGGTCCAGGA	This study
Comp2_Seep_symbiont_1	GTCAGTGTTGGCCCAGGA	This study
Comp3_Seep_symbiont_1	GTCAGTATTGGTCCAGGA	This study
L.mars_symb1	CTCTGCTGGATTCTGTCAAT	Zimmermann et al. [18]
L.mars1 + 2/helper1	GTCAAGGGTAGGTAAGGTTCTTCG	Zimmermann et al. [18]
L.mars1/helper2	CAGGCCCGAAGGCACTCCTGCAT	Zimmermann et al. [18]
EUB338I-III	GCTGCCTCCCGTAGGAGT GCAGCCACCCGTAGGTGT GCTGCCACCCGTAGGTGT	Daims et al. [33]
NON338	ACTCCTACGGGAGGCAGC	Wallner et al. [34]

The PFA-fixed tubeworm samples were dehydrated through serial incubations in 70% ethanol/PBS, 80% ethanol/PBS and 96% ethanol for 30 min. Embedding was done in Steedman's wax [35] by incubating the tissues in 96% ethanol/wax and three times in pure wax for 60 min at 38°C. The wax blocks were cut into 4 μm sections, mounted on Polysine-coated glass slides and incubated at 30°C overnight to improve adherence of the tissue sections. Slides were washed three times in 96% ethanol (5 min) and then rehydrated by incubation in 80% and 70% ethanol for 10 min. Endogenous peroxidases were inactivated by washing the slides in 0.2M HCl for 12 min. Permeabilization was performed by incubation in 20 mM Tris/HCl (pH 8) for 10 min, lysozyme solution (0.01 g/ml lysozyme, 0.05 M EDTA, 0.1 M Tris/HCl pH 8) for 30 min at 37°C and 20 mM Tris/HCl (pH 8) for 10 min. Sections were circled with a Pap-pen (Kisker Biotech, Steinfurt, Germany) to avoid leakage of the hybridization mixture across sections. CARD-FISH was performed according to [36] with modifications. 1 μl of each probe (50 ng/μl) was diluted in 150 μl hybridization buffer and then incubated for 3–24 hours at 46°C in dark humidity chambers. Slides were washed in pre-warmed washing buffer (48°C) and 1x PBS for 15 min before amplification. Amplification was done for 60 min at 46°C with Alexa-488 labeled tyramides (Molecular Probes, Leiden, the Netherlands). Subsequently, slides were washed in 1x PBS for 15 min and Milli-Q water for 10 min. For double CARD-FISH four probe combinations were tested: Seep_symbiont_1 + EUB338I-III, EUB338I-III + Seep_symbiont_1, Seep_symbiont_1 + L_mars1 and L_mars1 + Seep_symbiont_1. The HRP enzyme of the first probe was inactivated by incubation in 0.5% H_2_O_2_ in methanol for 30 minutes prior to hybridization of the second probe. Amplification for the second probe was done with Alexa-594 labeled tyramides (Molecular Probes, Leiden, the Netherlands). After air-drying the tissue sections were counterstained with 4,6-diamidino-2-phenylindole (DAPI) for 8 min, washed in Milli-Q water for 2 min and then embedded for microscopy in Vectashield (Vector laboratories, Burlingame, CA, USA). Overview images of whole tissue sections were taken on an Olympus BX53 compound microscope (Olympus, Tokyo, Japan) equipped with an ORCA Flash 4.0 (Hamamatsu Photonics K.K, Hamamatsu, Japan) camera using a 20× Plan-Apochromat objective and the software cellSens (Olympus, Tokyo, Japan). Close-up images were taken on a Zeiss LSM 780 confocal laser-scanning microscope (Carl Zeiss, Jena, Germany) equipped with an Airyscan detector (Carl Zeiss, Jena, Germany) using a 63× Plan-Apochromat oil-immersion objective. For image acquisition the Zen-Black software (Carl Zeiss, Jena, Germany) was used. Details of the image acquisition settings can be found in Table 3. Raw pictures were further processed in Fiji [37].

**Table 3:** Microscopy acquisition settings.

	Olympus		Airyscan		
Fluorophore	Excitation (nm)	Emission (nm)	Excitation laser (nm)	Detection window (nm)	Laser intensity (%)
DAPI	387 / 11	LP^*^ 409	405	BP^*^ 420–480	1.8
Alexa 488	470 / 40	LP 500	488	BP 495–550	0.57
Alexa 594	562 / 40	624 / 40	561	LP 570	0.15

^*^BP = Bandpass, LP = Longpass

## Results

### Host cytochrome-c-oxidase subunit I (*COI*) sequencing

**Fig 2:**
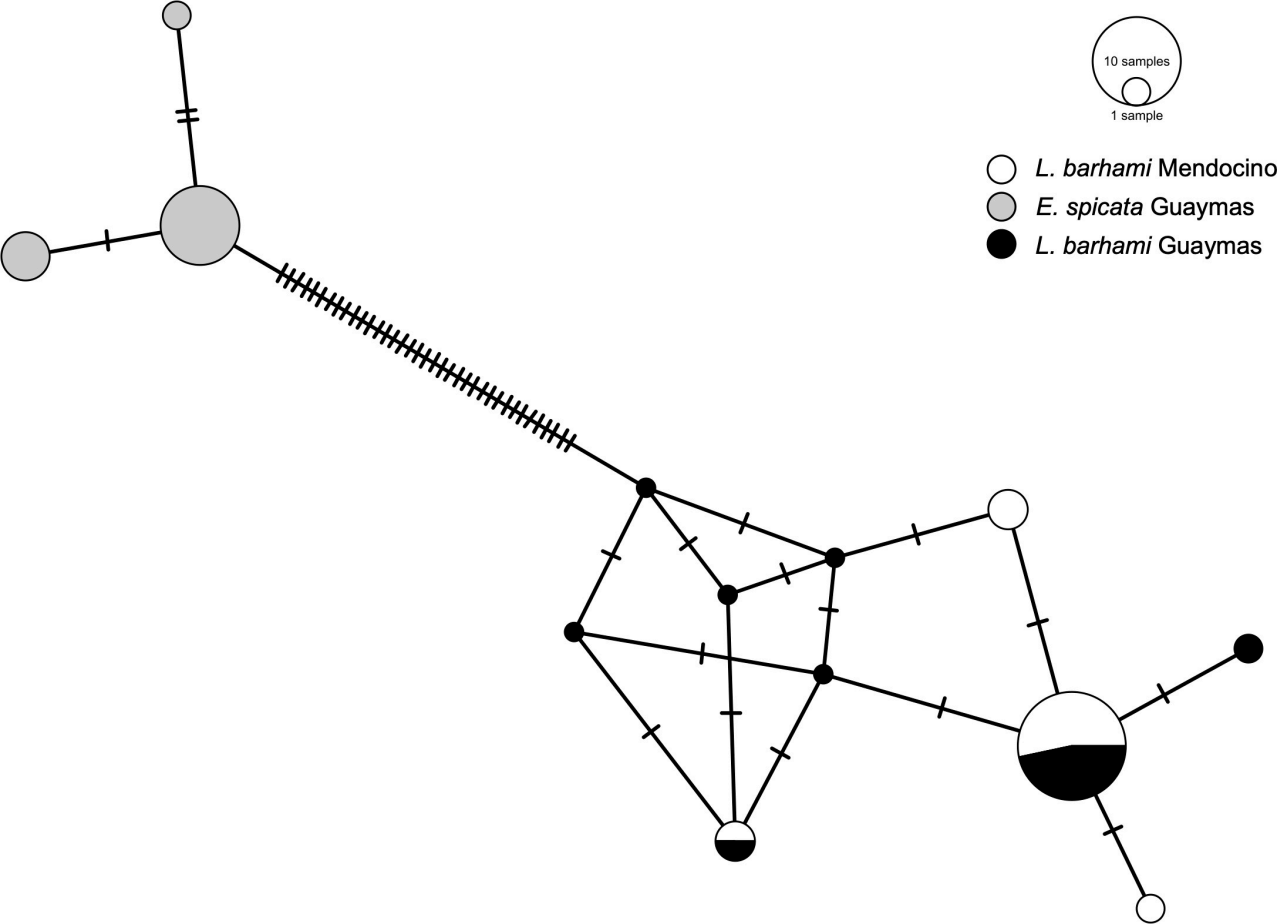
Haplotype network for the host mitochondrial *COI* sequences. Circles represent each one distinct haplotype, where size is proportional to frequency in the dataset. Lines on connecting branches indicate the number of mutations between different haplotypes.

Mitochondrial *COI* sequences revealed two highly divergent haplogroups that identified the two tubeworm species *E*. *spicata* and *L*. *barhami* (Fig 2). Each haplogroup consisted of one major haplotype and two to four minority haplotypes that differed only by a few mutations. For the two *L*. *barhami* populations no significant geographic structure could be detected as–with the exception of three site-specific sequences–haplotypes were shared among localities.

### *16S* amplicon sequencing and OTU clustering

*16S* amplicon sequencing resulted in an average of 73,133 raw paired-end reads per sample. After merging an average of 21,872 reads/sample remained. The USEARCH denoising pipeline identified three ZOTUs in the dataset (S2 Fig), two of which were excluded due to low abundance and/or occurrence in only a single sample, which is indicative of sequence artifacts or contaminants. Although one of these ZOTUs seemed to be related to an *E*. *spicata* endosymbiont, attempts to differentiate this and other sequences from the remaining dominant symbiont OTU with the OLIGOTYPING method [32] were unsuccessful as entropy values for each nucleotide position were much smaller than 0.2, implying that sequence polymorphisms were related to sequencing or PCR errors rather than biological variation (S1 Table). After all filtering steps and OLIGOTYPING analyses we could confirm only one tubeworm symbiont OTU–Seep symbiont 1 –that occurred in all samples independent of species or locality.

### CARD-FISH

**Fig 3:**
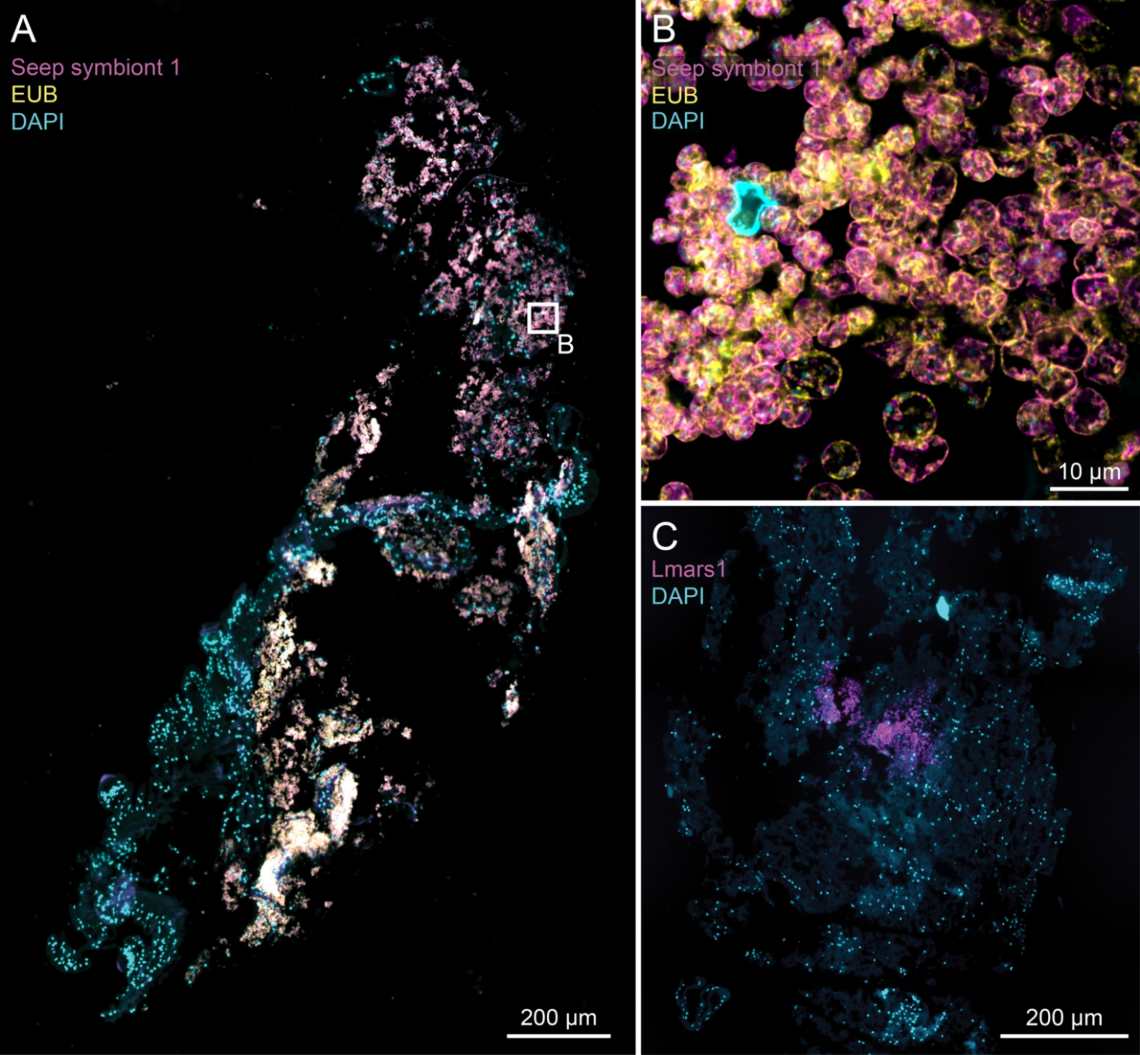
CARD-FISH images for seep endosymbionts in *L*. *barhami*. A) Overview image of a trophosome section that was double-stained with the Seep symbiont 1 probe (magenta) and the EUB338I-III probe (yellow). Both probes hybridize with the same symbiont, which creates a white fluorescence. Cyan dots indicate DAPI stain of host and symbiont DNA, while faint green coloring comes from autofluorescence of the host tissue. B) Close-up LSM image from the overview shown in picture A). Symbiont cells are coccoid with an approximate diameter of 5 μm. C) Trophosome tissue section stained with the Lmars1 probe (magenta) from Zimmermann et al. [18]. In contrast to the Seep symbiont 1 probe this probes only hybridized locally, suggesting the presence of additional phylotypes.

We investigated the presence of two symbiont phylotypes (targeted by the Seep symbiont 1 and L_mars1 probes) in a total of 151 throphosome sections (4 μm section thickness) on 28 slides of three *L*. *barhami* and two *E*. *spicata* specimens (S2 Table; LB45: 113 sections; LB33: 7 sections; LB39: 7 sections; ES9: 11 sections; ES19: 13 sections). Our analyses generally confirmed the presence of Seep symbiont 1 in the trophosomes of all investigated *L*. *barhami* individuals as well as in one *E*. *spicata* individual (Fig 3A and 3B; S3A, S3B and S4 Figs; S2 Table). The Seep symbiont 1 cells are coccoid-shaped with a diameter of approximately 5 μm.

*L*. *barhami* sections examined using the Seep symbiont 1 probe produced a positive signal in 56 of 94 cases, and four out of 16 *E*. *spicata* sections produced a signal. All symbionts that hybridized with the Seep symbiont 1 specific probe were also stained by the general EUB338I-III probe in the double CARD-FISH analyses (Fig 3; S3 and S4 Figs; S2 Table). No signals were detected for the negative control probe NON338 (S5 Fig). Technical issues or poor quality of the tissue sections produced hybridization failures for 45 sections.

To further assess symbiont diversity in the trophosome we used the previously validated symbiont probe L_mars1 that was designed for the *16S-V6* region [18] on the same samples and tissue sections in both mono and double CARD-FISH. In six sections out of 40 this probe stained localized aggregations of symbiont cells in the *L*. *barhami* trophosome, indicating the presence of at least one other seep symbiont phylotype that was not observed in the *16S* OTU set (Fig 3C; S3C Fig; S6 Fig; S2 Table). Similar to Seep symbiont 1 and in agreement with the results by Zimmermann et al. [18] for peripheral symbiont cells, this phylotype had a coccoid morphology with a cell diameter of ~5 μm. As positive CARD-FISH signals for this second phylotype were only observed in consecutive sections or sections within close proximity, we suppose that this symbiont was compartmentalized in a specific region of the trophosome in concordance with previous descriptions [18]. The repeated observation of these colonization aggregates and the differences in target sequences between symbiont phylotypes further indicate that these patterns are a true signal and do not result from unspecific binding of the L_mars1 probe (see also S1 Fig). No other hybridizations with this probe exhibited fluorescence. In 22 of the 40 sections a lack of hybridization signal was most likely observed because the symbiont type was not present in the investigated tissue sections. In these cases, a fluorescence signal was observed for the Seep symbiont 1 or EUB338I-III probes either in the same (for double CARD-FISH) or in adjacent sections (for mono CARD-FISH), indicating that the overall CARD-FISH experiment was successful. In the remaining 12 sections the hybridizations failed. These analyses were all double CARD-FISH analyses, which can fail at various steps due to e.g., insufficient binding or competition between probes, ineffective amplification reactions of the HRP, removal of probes during consecutive washing steps etc. We also tried the L_mars1 probe on three *E*. *spicata* slides (three sections), but did not obtain any fluorescence. Since in these cases positive signals were obtained for the EUB338I-III probe, we assume that this symbiont type was absent in the investigated sections (S2 Table). In summary these outcomes support the presence of a second, highly localized phylotype in the investigated *L*. *barhami* individual, which is consistent with previous findings in *L*. *anaximandri* [18].

## Discussion

Horizontal transmission presumably occurs during a narrow window of susceptibility, when locally occurring stages of symbiotic bacteria infect the settling tubeworm larvae [9]. Compared to vertical transmission, which leads to bottleneck effects and co-speciation in the symbiont population, the environmental infection mode is expected to result in symbiont heterogeneity within host individuals and the absence of specificity between a particular host and a particular symbiont [6, 11, 12]. In vestimentiferan tubeworms most traditional *16S* sequence analyses have shown that every tubeworm individual contains only one gammaproteobacterial symbiont phylotype, whereas evidence for host specificity has been inconclusive [2, 6, 13]. A limited number of studies challenged the finding of genetic homogeneity in the symbiont population. For instance, *16S* clone libraries showed that *Lamellibrachia anaximandri* from the Mediterranean Sea [18] as well as *Escarpia laminata* and *Lamellibrachia* sp. 2 from the Gulf of Mexico [17] can harbor two distinct symbiont phylotypes. Given that conventional PCR-based approaches are biased towards the most abundant DNA template, Zimmermann and colleagues [18] argued that these data likely underestimate the true degree of symbiont heterogeneity and that improved molecular analyses would be needed to identify the level of hidden diversity. To address these issues, we used high-throughput *16S* amplicon sequencing in co-occurring populations of the tubeworm species *L*. *barhami* and *E*. *spicata* from the eastern Pacific Ocean.

In contrast to Zimmermann et al.’s expectations our OTU clustering results revealed the presence of only one symbiont phylotype–Seep symbiont 1 –in *L*. *barhami* and *E*. *spicata*, which confirms previous notions that the tubeworm symbiosis is highly selective [6]. Although uptake of multiple strains would provide the advantage of selecting symbionts that are optimally adapted to the local environment, increased discrimination for a symbiotic partner might reduce the risk of infections by bacteria that do not provide any benefit to the host, given that horizontal transmission favors the evolution of cheaters [6]. The molecular mechanisms that underlie symbiont selectivity in vestimentiferan tubeworms are poorly understood. Perez and Juniper [20] hypothesized that the type-VI secretion system, found in the *Ridgeia* symbionts in their study, might be involved in partner choice as it is in the rhizobium-legume symbiosis [38]. Other types of secretion systems that might have similar roles in host selectivity have recently been discovered in the draft symbiont genomes of the seep tubeworms *Lamellibrachia*, *Escarpia* and *Seepiophila* [19, 21]. Evidence from gene expression analyses further suggests that the host immune system interacts directly with the symbionts to govern cell growth, as transcripts for peptidoglycan recognition proteins and toll-like receptors were significantly upregulated in the trophosome compared to symbiont-free tissues [39].

Despite the high selectivity observed in the tubeworm symbiosis relative to general microbial communities, our results imply a lack of specificity between host species and symbiont phylotype, given that Seep symbiont 1 associated with both *L*. *barhami* and *E*. *spicata*. These data agree with a previous study by Vrijenhoek et al. [15], which showed that co-occurring *E*. *spicata* and *L*. *barhami* from the Gulf of California contain the same *16S* phylotype–a remarkable finding because these host species belong to phylogenetically very distantly related vestimentiferan taxa [12]. A limited number of studies provided evidence for host-symbiont specificity in other co-occurring tubeworm species, although these investigations usually included comparisons of a typical seep species with a typical vent species [12]. Vent tubeworms such as *Riftia pachyptila* acquire their symbionts from the surrounding water during a short period between larval settlement and metamorphosis to the juvenile stage [9]. The mechanisms of symbiont acquisition and durations of infection susceptibility have not been similarly investigated in seep tubeworm taxa, which might influence their symbiont compositions relative to those of their vent counterparts. In contrast to vent tubeworms seep tubeworms possess long body extensions called 'roots' [40], with which they obtain sulfide from the seafloor sediments for their symbionts. Cordes et al. [41] suggested that the root system plays other important roles in the tubeworm symbiosis. They hypothesized that seep tubeworms use their roots to pump sulfate byproducts into the surrounding sediments, where it is consumed during sulfate-driven anaerobic methane oxidation. This, in turn, would produce sulfide that the tubeworm can deliver to its endosymbiont. Perhaps the root system has more far reaching functions and continuously acquires new symbiotic bacteria, as it appears to do in bone-eating *Osedax*, a related siboglinid tubeworm [23, 42] and in the legume-rhizobia symbiosis [43]. Such potential differences in symbiont uptake mechanisms could be alternative explanations for the observed differences in symbiont compositions between vent and seep tubeworms without the presence of genetic specificities.

Disentangling the roles of phylogenetic, genetic, physiological, behavioral and ecological factors affecting the acquisition and enrichment of symbiont phylotypes by various tubeworm species will be difficult. One limitation of our study is that we only investigated a small fragment of the evolutionarily conserved *16S* rRNA gene, which might not have sufficient resolution to reveal patterns of symbiont specificity and variability if they exist. Metagenomic studies in the deep-sea hydrothermal vent mussel *Bathymodiolus septemdierum* have recently shown that this species harbors a single endosymbiotic phylotype with a monomorphic *16S* rRNA sequence, but that the population of this phylotype varies significantly in the composition of key metabolic genes for hydrogen oxidation and nitrate reduction [44]—a crucial finding that helps to understand how host animals might adapt to the dynamic environmental conditions at vents and which remained undetected by previous methods. Interestingly, we observed another seep symbiont phylotype when targeting a different region of the *16S* rRNA with CARD-FISH. This result could indicate that the *V4* region might not be an appropriate target to investigate diversity in tubeworm endosymbionts, at least in this sample set. Studies on pathogenic bacteria have previously shown that the *V4* region can be more conserved than other hypervariable regions [45]. Our findings are still unexpected given that minor variations in the *V4* regions can be found between publicly available seep symbiont *16S* sequences (e.g., GenBank). One other limitation of our study could be that we investigated only adult tubeworms but not larval stages in which the symbionts are acquired. For example, it is possible that multiple infections occur in tubeworm larvae, but that different symbiont types outcompete each other during the development of the tubeworm or that some strains are lost due to drift and only a subset of the initially acquired symbiont diversity is retained. PCR-free metagenome analyses in a sufficient number of samples and developmental stages might be best suited for further research that intends to address these concerns and uncover symbiont diversity and specificity in vestimentiferan tubeworms that might be hidden below the *16S* rRNA level.

## Supporting information

S1 Fig **Target *16S* rRNA regions and specificity of CARD-FISH probes targeting the (A) Seep symbiont 1 and (B) L_mars1 phylotypes.** Sample Sanger reference sequences for the *L*. *anaximandri* symbiont type A (KC) from the Mediterranean Sea and the *L*. *barhami* symbiont (AY) from various geographic regions were downloaded from GenBank. Numbers in front of the sequences indicate the nucleotide position in the *16S* rRNA alignment.
(PDF)

S2 Fig Abundances for ZOTUs in the unfiltered dataset. Only the dominant *L*. *barhami* symbiont phylotype could be independently verified with OLIGOTYPING and CARD-FISH analyses.
(PDF)

S3 Fig CARD-FISH images for seep endosymbionts in *L*. *barhami* as shown in Fig 3 of the manuscript. Greyscale images are added to help identify the individual signals in the overlay image, which indicate that both probes bound to the same regions even though intensities differ in certain areas.
(TIF)

S4 Fig Additional CARD-FISH images for Seep symbiont 1. Overview images (A and B) are from different trophosome sections of *L*. *barhami* individual 45. The sections were double-stained with the Seep symbiont 1 probe (magenta) and the EUB338I-III probe (yellow). Both probes hybridize with the same symbiont, which creates an orange to white fluorescence. Cyan dots indicate DAPI stain of host and symbiont DNA, while faint green coloring comes from autofluorescence of the host tissue. The grey scale images represent each individual channel.
(PDF)

S5 Fig Negative control CARD-FISH image of a *L*. *barhami* trophosome section that was adjacent to the ones in Fig 3A and 3B. Hybridization was performed with the NON338 probe using Alexa-488 (magenta) and Alexa-594 (yellow) tyramides. Image processing was done with the same settings as in Fig 3. No signals other than green autofluorescence are visible indicating that the probe did not bind. Cyan dots are DAPI stains of symbiont DNA and host nuclei.
(PDF)

S6 Fig Additional CARD-FISH images for symbiont cells stained with the Lmars1 probe. Overview images (A–C) are from consecutive trophosome sections of *L*. *barhami* individual 45, indicating the localized aggregation of this symbiont phylotype. The aggregation was always in the same location of the trophosome. Zoom in images (D–G) show the coccoid morphology (diameter ~ 5 μm) of these cells.
(PDF)

S1 Table *16S* rRNA sequence and site-specific entropy values of symbiont sequences as identified by the USEARCH and OLIGOTYPING methods. The entropy values for each nucleotide position are below 0.2, which indicates that observed variation in this sequence is related to sequencing or PCR errors.
(XLSX)

S2 Table Summary of mono and double CARD-FISH results for each slide investigated. Most analyses were done in *L*. *barhami* individual #45 as the trophosome tissue was optimally preserved. Positive signals for the Seep symbiont 1 (Ssym1) and EUBI-III probes were observed in well preserved sections of other *E*. *spicata* and *L*. *barhami* individuals. Results exclude initial tests to identify the correct formamide concentration for the different probes. The EUBI-III and NON probes were not used in all slides after establishing that they consistently provided positive and negative signals, respectively.
(XLSX)
