# Supplementary material for: Intra-host symbiont diversity in eastern Pacific cold seep tubeworms identified by the 16S-V6 region, but undetected by the 16S-V4 region
Source: PLoS One. 2020 Jan 15;15(1):e0227053. doi: 10.1371/journal.pone.0227053 (PMC6961877; doi:10.1371/journal.pone.0227053)
Supplement: S1 Fig — Target 16S rRNA regions and specificity of CARD-FISH probes targeting the (A) Seep symbiont 1 and (B) L_mars1 phylotypes. Sample Sanger reference sequences for the L. anaximandri symbiont type A (KC) from the Mediterranean Sea and the L. barhami symbiont (AY) from various geographic regions were downloaded from GenBank. Numbers in front of the sequences indicate the nucleotide position in the 16S rRNA alignment. (PDF) [file pone.0227053.s001.pdf]

A

```
Ssym1_probe      711 --TCCTGGACCAACACTGAC--
KC832741.1       711 CTTCCTGGACCAGCACTGACGC
KC832740.1       711 CTTCCTGGACCAGCACTGACGC
KC832738.1       711 CTTCCTGGACCAGCACTGACGC
KC832735.1       711 CTTCCTGGACCAGCACTGACGC
KC832733.1       711 CTTCCTGGACCAGCACTGACGC
KC832731.1       711 CTTCCTGGACCAGCACTGACGC
AY129103.1       711 CTTCCTGGACCAACACTGACGC
AY129094.1       711 CTTCCTGGACCAACACTGACGC
AY129093.1       711 CTTCCTGGACCAACACTGACGC
AY129113.2       711 CTTCCTGGACCAACACTGACGC
AY129091.1       711 CTTCCTGGACCAACACTGACGC
AY129090.1       711 CTTCCTGGACCAACACTGACGC
```

KC832741.1–KC832731.1: *L. anaximandri* symbiont type A

AY129103.1–AY129090.1: *L. barhami* symbiont

B

```
Lmars1_probe     972 --ATTGACAGAATCCAGCAGAG--
KC832741.1       972 ACATTGACAGAATCCAGCAGAGAT
KC832740.1       972 ACATTGACAGAATCCAGCAGAGAT
KC832738.1       972 ACATTGACAGAATCCAGCAGAGAT
KC832735.1       972 ACATTGACAGAATCCAGCAGAGAT
KC832733.1       972 ACATTGACAGAATCCAGCAGAGAT
KC832731.1       972 ACATTGACAGAATCCAGCAGAGAT
AY129103.1       972 ACATTGACAGAATCCTGTAGAGAT
AY129094.1       972 ACATTGACAG-ATCCTGTAGAGAT
AY129093.1       972 ACATCGACAGAATCCTGTAGAGAT
AY129113.2       972 ACATCCTCAGAACTTGTTAGAGAT
AY129091.1       972 ACATCCTCAGAACTTGTTAGAGAT
AY129090.1       972 ACATCCTCAGAACTTGTTAGAGAT
```

KC832741.1–KC832731.1: *L. anaximandri* symbiont type A

AY129103.1–AY129090.1: *L. barhami* symbiont
