# Supplementary figures and images for: Intra-host symbiont diversity in eastern Pacific cold seep tubeworms identified by the 16S-V6 region, but undetected by the 16S-V4 region
Source: PLoS One. 2020 Jan 15;15(1):e0227053. doi: 10.1371/journal.pone.0227053 (PMC6961877; doi:10.1371/journal.pone.0227053)

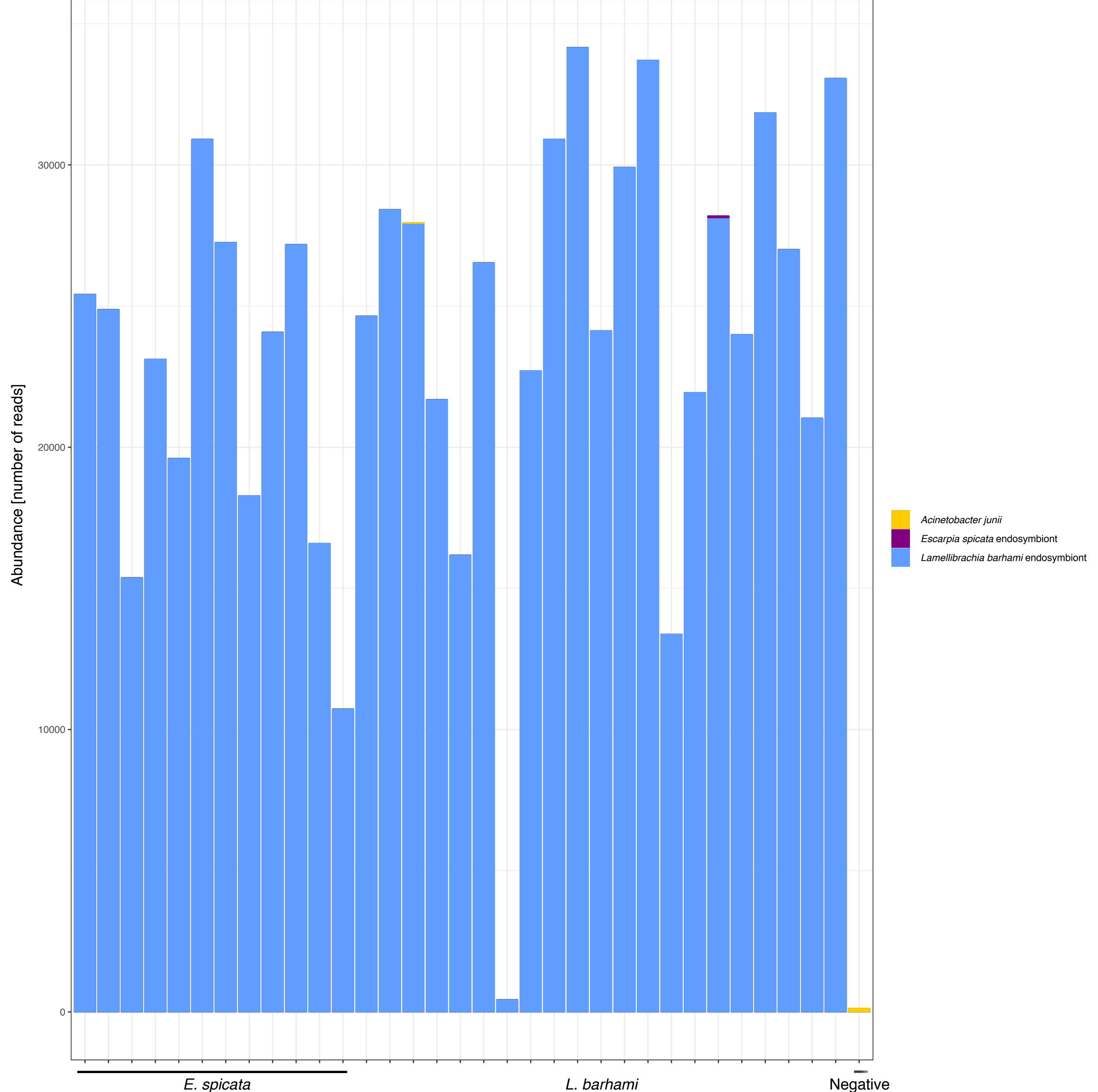

Supplement: S2 Fig — Only the dominant L. barhami symbiont phylotype could be independently verified with OLIGOTYPING and CARD-FISH analyses. (PDF) [file pone.0227053.s002.pdf]

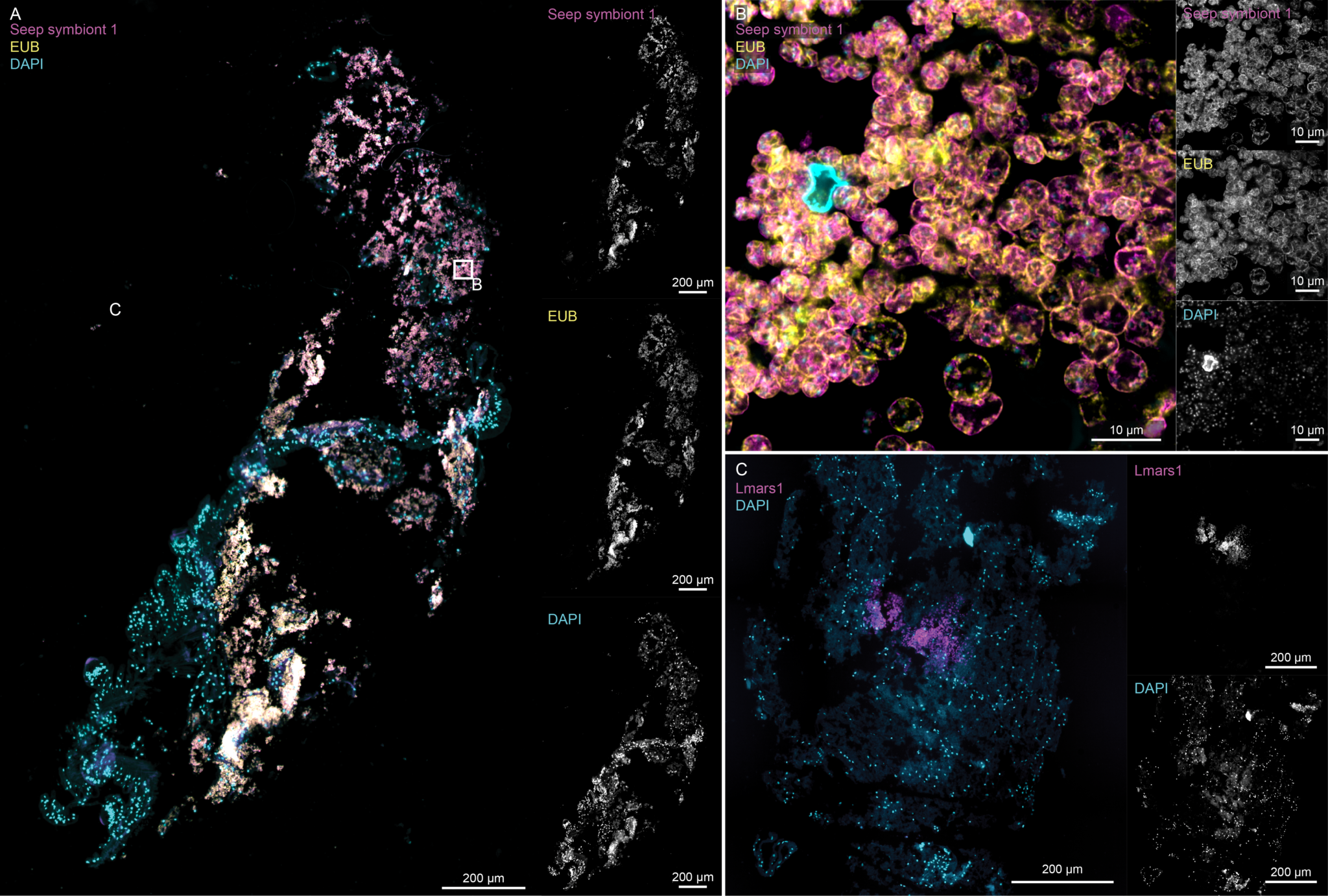

Supplement: S3 Fig — Greyscale images are added to help identify the individual signals in the overlay image, which indicate that both probes bound to the same regions even though intensities differ in certain areas. (TIF) [file pone.0227053.s003.tif]

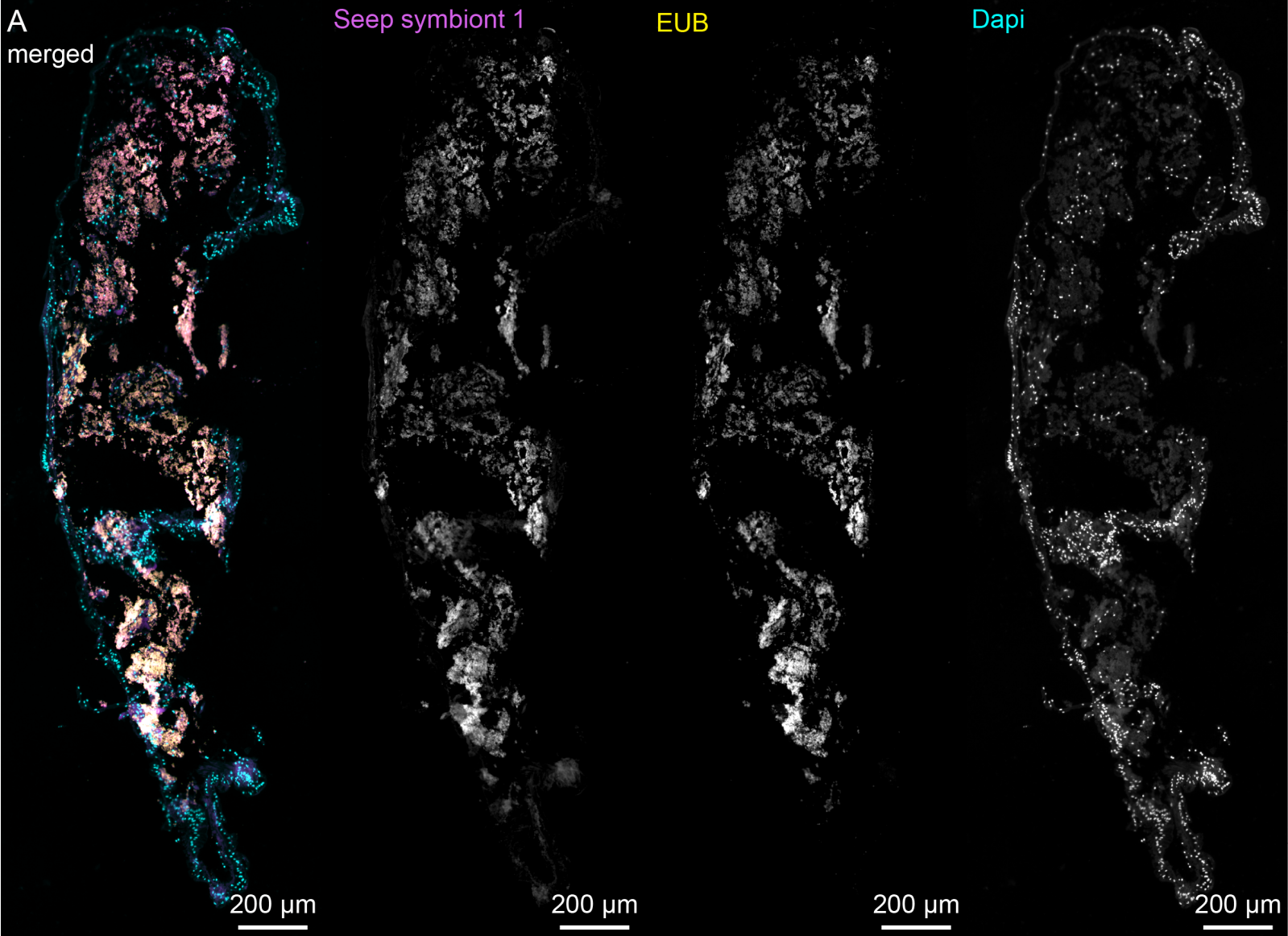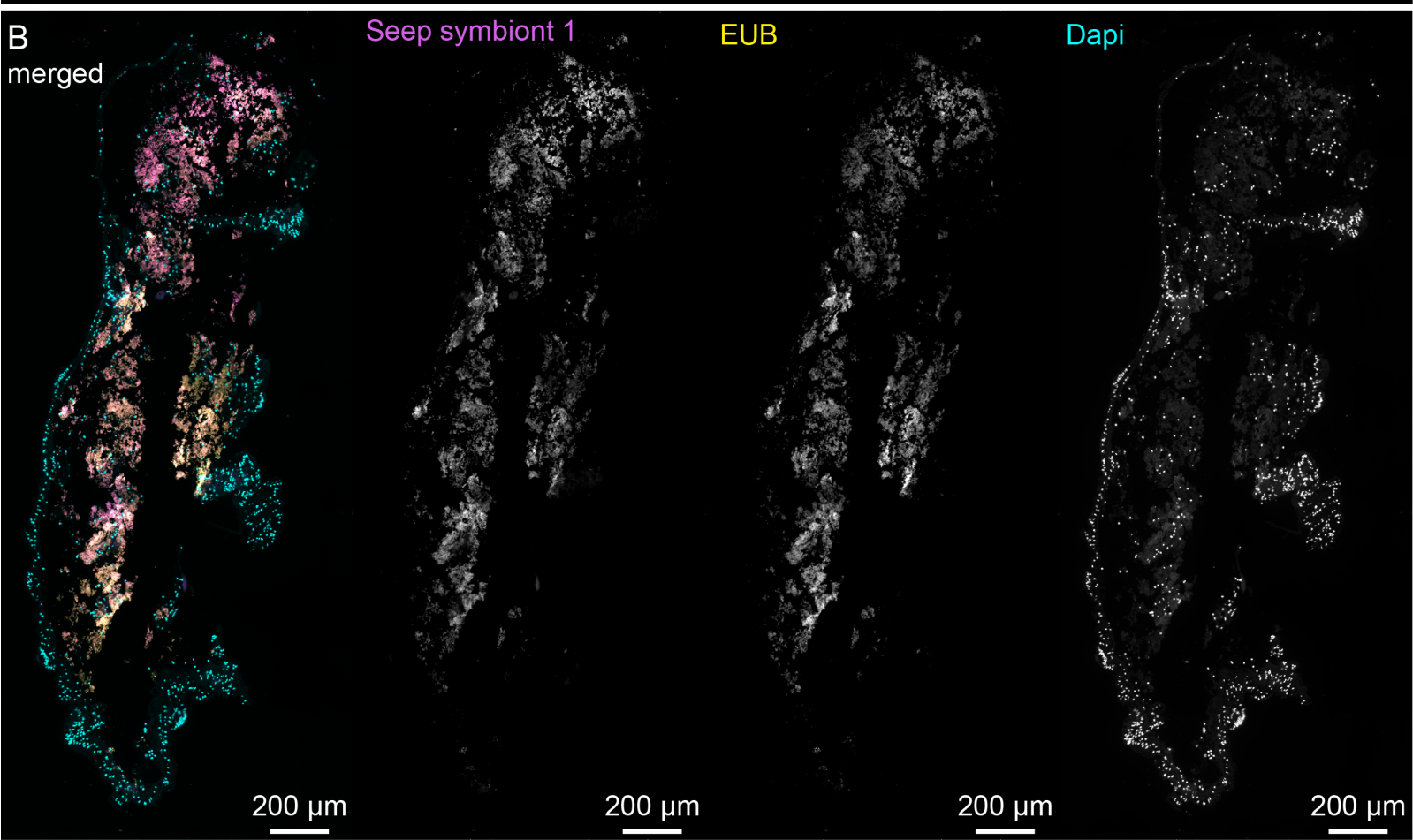

Supplement: S4 Fig — Overview images (A and B) are from different trophosome sections of L. barhami individual 45. The sections were double-stained with the Seep symbiont 1 probe (magenta) and the EUB338I-III probe (yellow). Both probes hybridize with the same symbiont, which creates an orange to white fluorescence. Cyan dots indicate DAPI stain of host and symbiont DNA, while faint green coloring comes from autofluorescence of the host tissue. The grey scale images represent each individual channel. (PDF) [file pone.0227053.s004.pdf]

Non 338 594 nm  
Non 338 488 nm  
DAPI

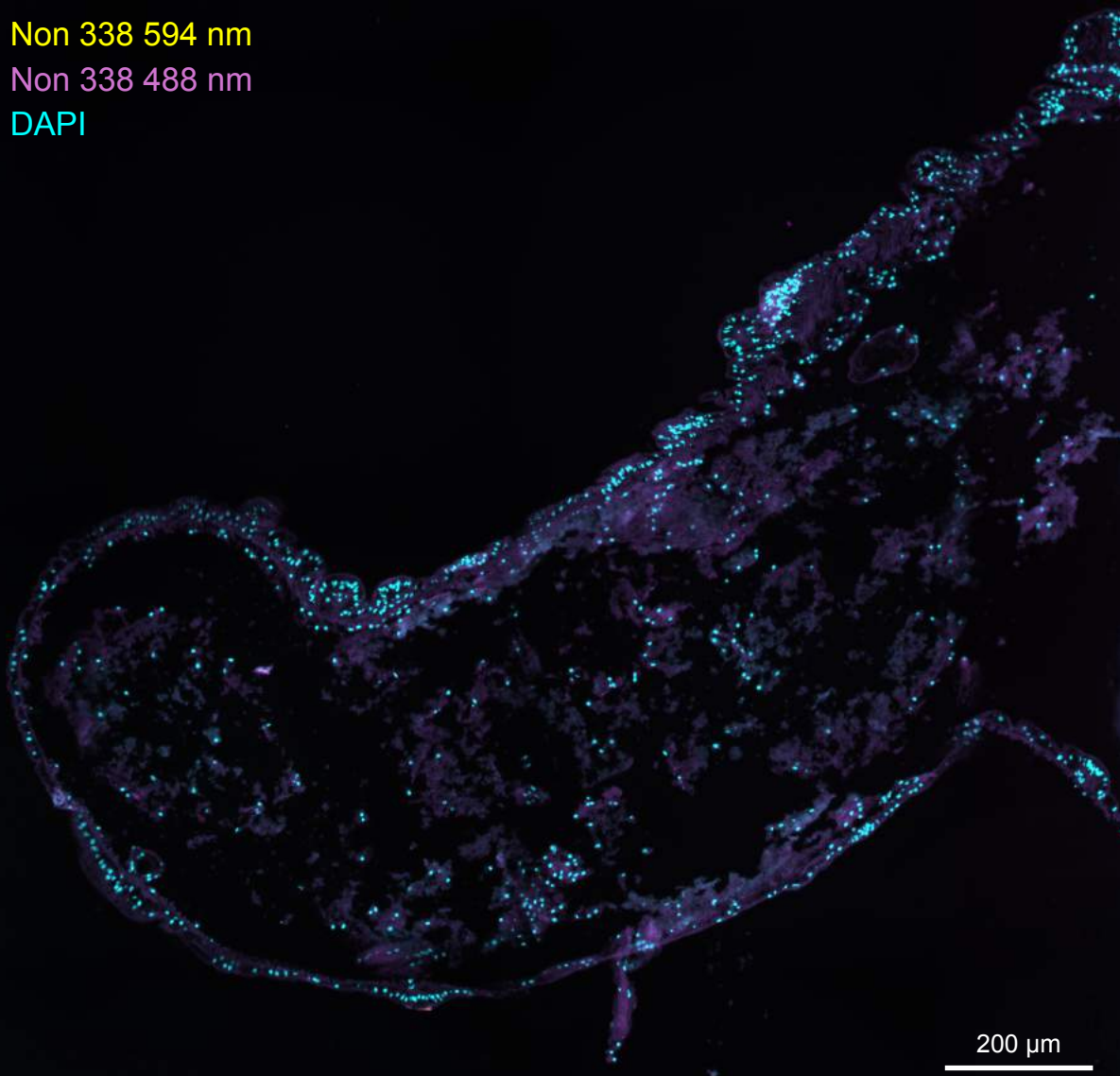

Non 338 594 nm

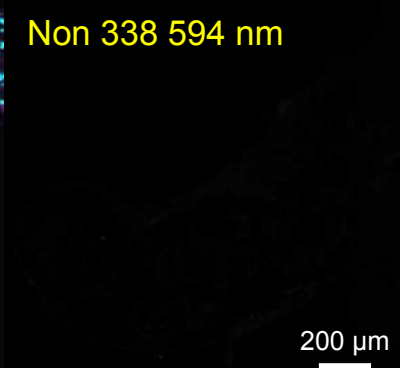

Non 338 488 nm

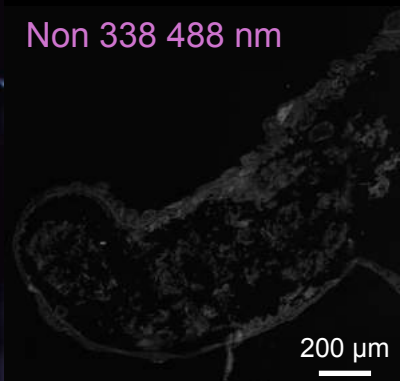

DAPI

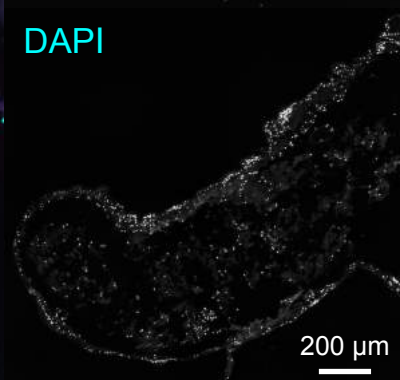

Supplement: S5 Fig — Hybridization was performed with the NON338 probe using Alexa-488 (magenta) and Alexa-594 (yellow) tyramides. Image processing was done with the same settings as in Fig 3. No signals other than green autofluorescence are visible indicating that the probe did not bind. Cyan dots are DAPI stains of symbiont DNA and host nuclei. (PDF) [file pone.0227053.s005.pdf]

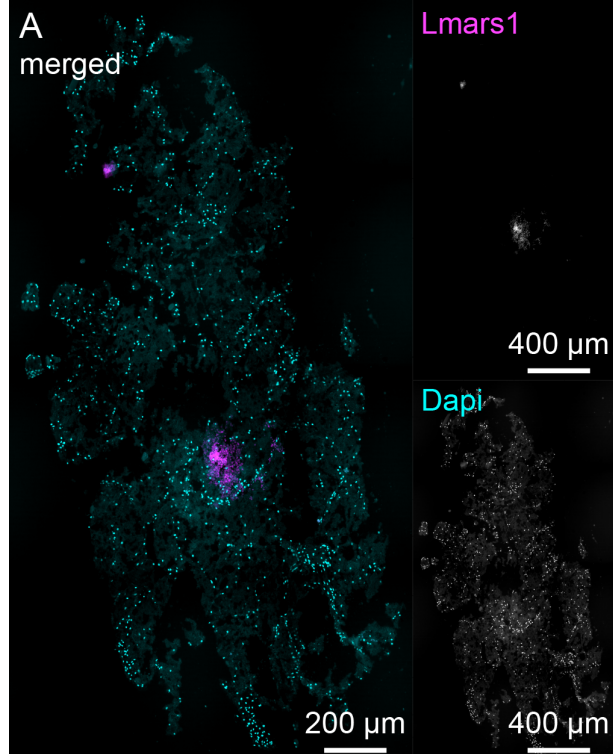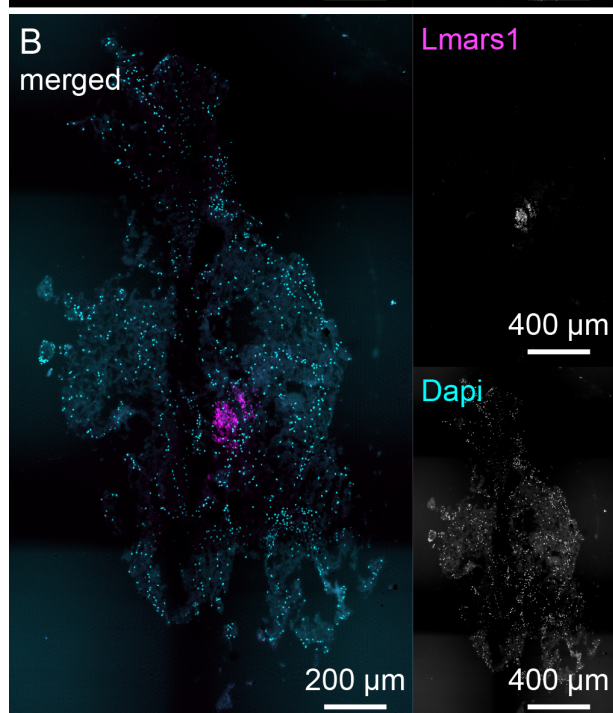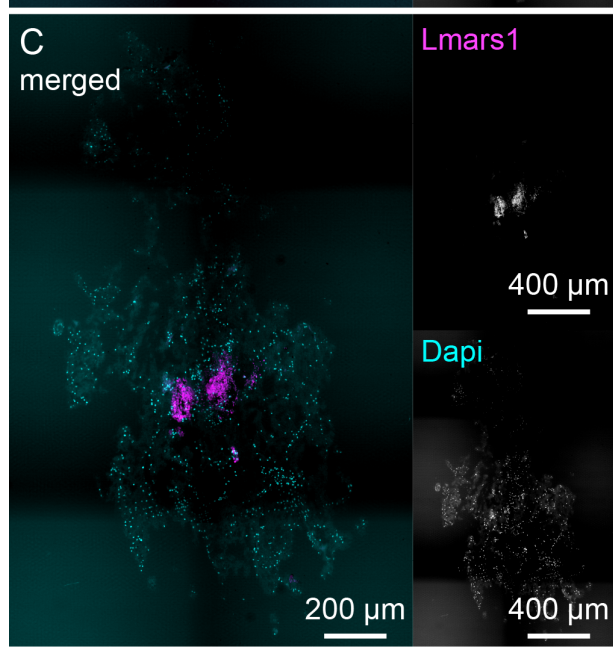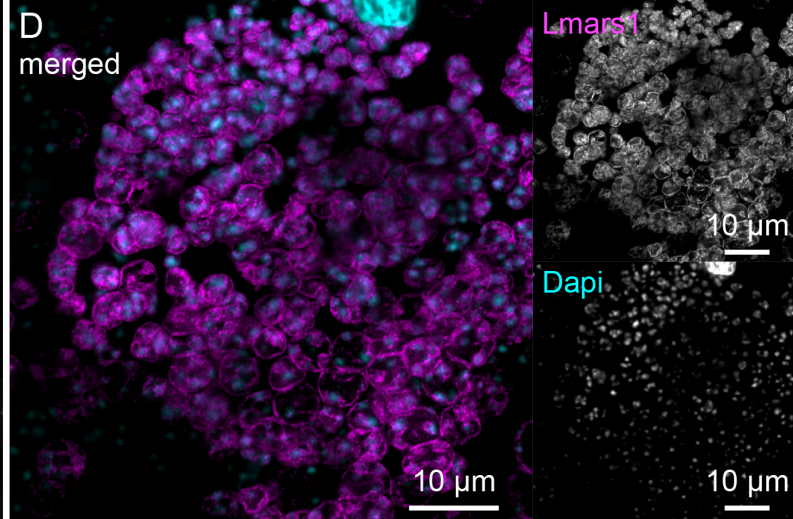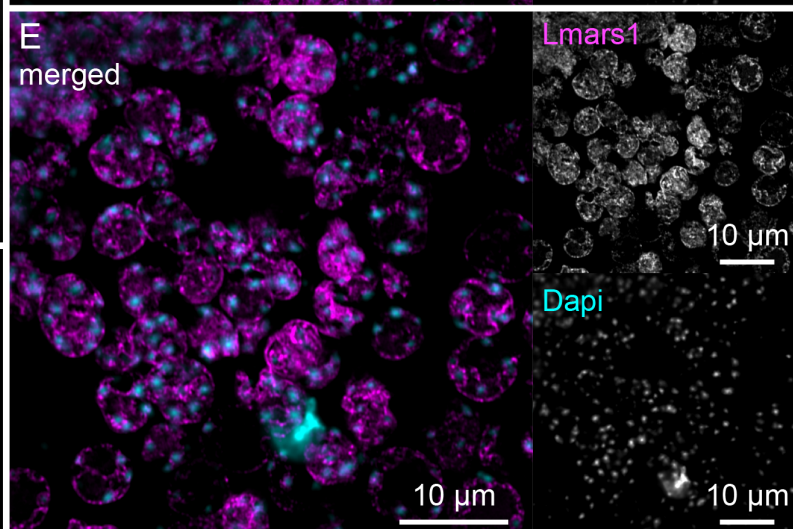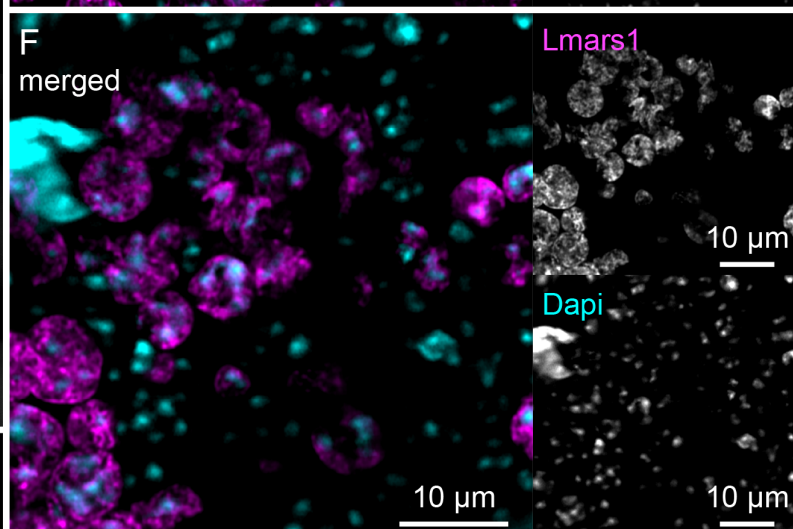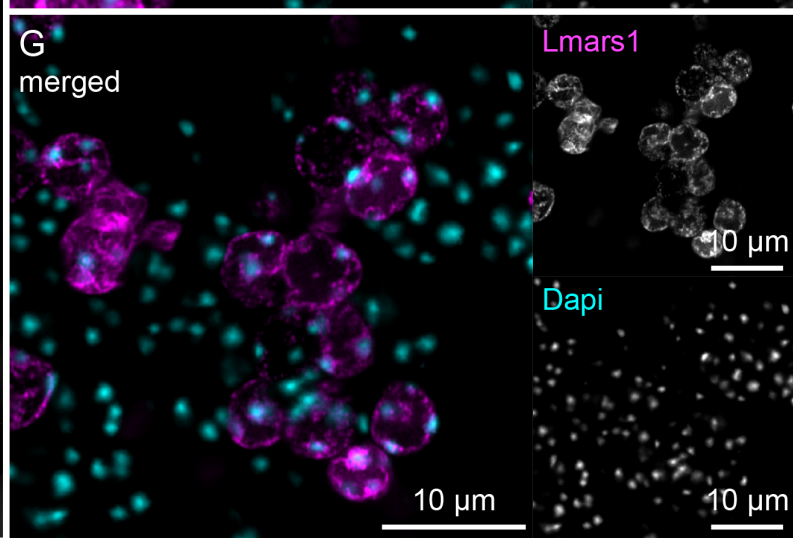

Supplement: S6 Fig — Overview images (A–C) are from consecutive trophosome sections of L. barhami individual 45, indicating the localized aggregation of this symbiont phylotype. The aggregation was always in the same location of the trophosome. Zoom in images (D–G) show the coccoid morphology (diameter ~ 5 μm) of these cells. (PDF) [file pone.0227053.s006.pdf]
